# User violence prevention and intervention measures to minimize and prevent aggression towards health care workers: A systematic review

**DOI:** 10.1016/j.heliyon.2023.e19495

**Published:** 2023-09-01

**Authors:** Paloma López-Ros, Reyes López-López, David Pina, Esteban Puente-López

**Affiliations:** a Department of Behavioral Sciences and Health, University Miguel Hernández, Elche, Spain; b Applied Psychology Service (SEPA), University of Murcia, Murcia, Spain; c Department of Socio-Sanitary Sciences, University of Murcia, Murcia, Spain; d Department of Psychology, Universidad de Almería, Almería, Spain

**Keywords:** Violence, Primary care, Users, Healthcare workers, Systematic review

## Abstract

Workplace violence in the health care setting is a social problem of great interest both at the health care level and in research in recent decades. The most common type of violence is the one coming from the user towards the professional. Although the bibliography includes multiple preventive actions focused on working with professionals, there are hardly any studies that explore and collect actions aimed at the user. The aim of this study is to analyze the results of the literature to provide an overview of the current evidence. Specifically, it aims to describe the various user-directed strategies or interventions aimed at reducing workplace violence experienced by professionals within the healthcare sector. A systematic review was performed following the Preferred Reporting Items for Systematic Reviews and Meta-Analyses (PRISMA), methodology of publications published up to December 2022 in the main databases. Studies that met the previously established eligibility criteria were identified. A peer review of the risk of bias was performed and the data were extracted from a previously elaborated template. The search yielded 5231 articles of which 11 were finally included in the review. Of these, 3 had a quantitative design, 7 had a qualitative design and one had a combined design. Of these, 38 measures or actions aimed at the user were compiled, grouped into four blocks according to the attitudinal objective pursued: Improvement of communication and creation of links, involvement of the user in joint decisions with the staff, informing and training the user, and other independent proposals. This study makes it possible to explore actions aimed at users with the objective of reducing violence towards health professionals. It collects and makes available to the scientific community a set of measures aimed at making a change of attitude in the perpetrator themselves, with the involvement of the perpetrator in the health system. This set of collected measures provides researchers with a basis to be taken into account for the implementation of future prevention plans according to the new multicomponent prevention models and with the involvement of the perpetrator themselves.

## 1 Introduction

Violence in the work environment is a social problem of growing interest in both research and professional practice. However, there is still no scientific consensus on a definition of workplace violence [1]. Healthcare workers may be subjected to workplace violence [2] although exposure in other contexts has also been observed in this occupational group [3]. The concern to solve this problem is international and it is necessary to invest resources and carry out prevention campaigns and programs to improve occupational health and safety [4].

The International Labour Organization (ILO) defines violence in the workplace as “any action, incident or behaviour that departs from reasonable conduct in which a person is assaulted, threatened, harmed, injured in the course of, or as a direct result of, his or her work” [5]. The Occupation Health and Safety Act [6] also defines it as “the exercise of physical force by a person against a worker, in a workplace, that causes or could cause physical injury to the worker; a statement or behaviour that it is reasonable for a worker to interpret as a threat to exercise physical force against the worker, in a workplace, that could cause physical injury to the worker”.

Whether there is a consensus to include in the official definitions of workplace violence behaviors that include physical aggression, verbal abuse and harassment. Among the most common physical manifestations are hitting, kicking, slapping, biting and pushing and/or pinching, among others. Psychological injuries range from threats, abuse of power, verbal abuse, mobbing and offensive behaviors with retaliatory or repetitive intent [7,8].

Workplace violence has been increasing in recent decades on an international scale [7]. The healthcare setting is one of the fields in which the greatest number of assaults occur, with almost a quarter of workplace violence occurring in this sector [[5], [6], [7], [8]]. It has recently been estimated that the overall prevalence of workplace violence in the healthcare sector is 58.7% [9], However, this percentage is reported to range from 2.1 to 36.1% in Europe to 46.0–71.4% in North America [10]. Although these data could be even higher since there are several factors that influence the prevalence data collected, both the health professionals who can come to take violence as a link to their work [11]. Health professionals express fear of reprisals and high levels of bureaucracy, which leads to underreporting of violent acts due to the lack of complaints and records [[12], [13], [14], [15], [16], [17], [18], [19], [20], [21], [22], [23]]. One possible explanation for underreporting is based on the fact that healthcare personnel may come to think that workplace violence is normal and therefore do not report it. Healthcare workers consider workplace violence to be normal [2]. Of the existing records, healthcare personnel suffer a greater proportion of non-physical violence (42.5%) than physical violence (20.8–24.4%). Among the most frequent manifestations of non-physical violence, verbal violence (57.6% and 66.8%), threats (33%) and sexual harassment (10.5–12.4%) have been observed [9,10]. In Spain, the systematic review by Serrano-Vicente et al. [14] on assaults on healthcare workers concludes that the prevalence ranges between 58% and 80%. A distinction was also made between physical and verbal violence, with verbal violence being more common than physical violence.

Specifically, the prevalence of violence in the healthcare sector also varies depending on the service where the professional practice is carried out, with Emergency, Primary Care or Mental Health services registering a higher rate of violent acts [10,[15], [16], [17], [18]]. This rate appears to have increased since the onset of the Coronavirus Disease 2019 (COVID-19) pandemic due to the high demand and burden of care [[19], [20], [21]]. In a recent study of hospital emergency departments, 100% of those surveyed acknowledged having suffered at least once from workplace violence in the last year [18]. Nurses and medical staff are among the most frequently assaulted health care professionals, due to their close and frequent contact with patients and their families [1,22,23]. Some studies affirm that non-health personnel are affected to the same or greater extent than health personnel, especially administrative personnel [18]. Violence in this context can come from people outside the health service (patients and/or their companions) as well as from people in the service itself (coworkers or supervisors), although it is more common to see violence exercised by patients and their companions [24,25]. The high rates of violence suffered by healthcare personnel, coupled with the low to medium effectiveness of prevention plans, means that workplace violence in healthcare continues to be of great interest to the public [4].

There are different factors that can lead the user and their family members to cause conflictive situations, and there may even be a combination of several factors. Among the different reasons, the literature points out Gender, diagnosis, symptomatology, environmental conditions, perception of poor communication, substance abuse, feelings of frustration, denial of services, overcrowded wards or staff training [13,25]. Repeated exposure to workplace violence can have multiple negative consequences for the health professional. These consequences can involve psychological manifestations and even lead to physical manifestations. Professional burnout, post-traumatic stress, anxiety, sleeping difficulties, decreased job satisfaction and stress, among others, are frequently found [23]. In addition, the services themselves also suffer collateral consequences such as increased costs due to absenteeism and staff leave, the number of occupational accidents or a decrease in the quality of care, which in turn can lead to an increase in the number of violent behaviors towards health professionals [23].

Within this context, different studies have been designed with the aim of designing and implementing strategies to prevent user violence [[26], [27], [28], [29], [30]]. Of the most recent systematic reviews, those by Somani et al. [1] and Geoffrion et al. [22] stand out, showing that multicomponent interventions that include training and practical training are effective in preventing workplace violence. It has been observed that, when users are part of the studies, they feel integrated when working together and a greater attachment to the health system, obtaining very positive results. In addition, the effectiveness of studies whose prevention programs are focused entirely on the personnel do not show positive data in terms of reducing violence. It is also necessary to focus prevention measures on the causal factor, in this case, the user and their family members [1,22].

For all these reasons, a synthesis of the literature on prevention/intervention programs to reduce violence in the health care work environment is considered necessary. A wide variability has been observed in this setting, i.e., the resources used for the different professionals in the health sector (medicine, nursing, auxiliary personnel) are not similar, nor are those used in the different units (emergency, primary care, psychiatry, etc.). This is why it is necessary to explore those points in common that allow professionals interested in the subject to explore the different approaches made for the prevention of violence in the healthcare environment, in this case, those aspects that work directly with users [1,2].

However, we have not found studies that synthesize the strategies that can be applied with the users themselves to prevent violence in health services. In order to provide evidence in this regard, the main objective of the present study was to carry out a systematic review of intervention programs to reduce violence by users against health care personnel. Specifically, we propose a review focused exclusively on those measures that involve the users themselves.

## 2 Methods

A systemic review of the scientific evidence was performed following the indications of the PRISMA guidelines [31]. The location where each item in the guidelines is reported in this article is available at Supplementary File 1: https://osf.io/dt2v4. This study was approved by the Research Ethics Committee of the University of the authors assigned the Code of the Office of Responsible Research (COIR) with ref. 220,426,115,743.

### 2.1 Search strategy

A systematic search of the literature published up to December 2022 was performed in the following databases: EBSCOhost (Academic Search Premier, Psychology and behavioral science collection, APA Psycharticles, APA PsychInfo, Medline, Education Source, ERIC, Violence and Abuse Abstracts y PsicoDOC), The core collection of Web of Science, ProQuest Central (PubMed, Social Services Abstracts y Sociological Abstracts) y Cochrane Library Plus (CENTRAL) (Embase, NIOSHTIC/NIOSHTIC-2, HSELINE, ISDOC, Scielo, Dialnet, CUIDEN, CINAHL, Scopus y Science Direct). The search terms were terms related to the health profession, units, aggression, and interventions. The complete search strategies followed in each of the databases are available at Supplementary File 2: https://osf.io/3ydh2. In addition, the references of the included studies were consulted to obtain additional articles and include them as an incidental bibliography. Similarly, the reference lists of all primary studies and articles were checked for additional references, and experts in the field were asked to identify additional unpublished materials.

No previous systematic review focused exclusively on user-directed measures was found, so no restriction was established on the years for the search, and no restriction was established by language. In order to reduce duplication and provide transparency to the review process, as well as to minimize information bias [32], this study was registered in PROSPERO (Prospective International Register of Ongoing Systematic Reviews, http://www.crd.york.ac.uk/prospero) from its beginning (Registration Nº: CRD42022290030).

### 2.2 Selection criteria

We included studies that: (a) included intervention and/or prevention programs and/or strategies aimed at reducing workplace violence by users or their families towards health care professionals; (b) included programs or program proposals focused on the health care user; (c) used randomized controlled trials (RCT), cluster randomized controlled trials (CRCT), controlled before and after studies (CBA) and/or qualitative studies as research design; and (d) had the full text of the study available.

On the other hand, we excluded those studies (a) that evaluated frequency, latency, duration, or recurrence of violence, but did not propose intervention or prevention plans; (b) articles that included intervention or prevention measures in workers and users of non-health care settings.

The intervention strategies included in this study include strategies or interventions that are mandatory or voluntary, in a single session or in several sessions, face-to-face, online or combined, as well as synchronous or asynchronous components, interventions or strategies carried out in or outside healthcare centers, as well as intervention and/or prevention programs and/or strategies aimed at reducing workplace violence among users with or without outcome measures, and taking into account independent programs or those carried out together with other interventions aimed at healthcare personnel.

### 2.3 Selection of studies

The selection of studies was performed by two investigators working independently in duplicate following the eligibility criteria (first and second author). First, titles and abstracts were reviewed, eliminating those that were clearly ineligible. These were coded as “selected” (eligible, potentially eligible, or uncertain) or “not selected”, with only those showing potential for selection proceeding to the second phase. In case of disagreement, these were discussed until agreement was reached. In the second phase, two researchers (first and second authors) independently read and reviewed the full-text articles after the second round of screening to decide which studies would be included. Duplicates were manually removed in both this and the previous phase. Again, they were coded as “selected” (eligible, potentially eligible, or uncertain) or “not selected”. Disagreements were resolved by consensus or by consulting a third person on the team (third author). The reason for the “not selected” papers was recorded. The authors in charge of this review have extensive experience in the field of health violence. Each excluded study was reviewed by another subgroup of authors with experience in the field to ensure its reliability (third and fourth authors).

### 2.4 Risk of bias assessment

Given that the included studies have some particularities that are not covered by any of the existing bias analysis guides, one of our own elaboration was carried out, taking as an example the Strengthening the Reporting of Observational Studies in Epidemiology (STROBE) initiative statement for observational studies [34], available at Supplementary File 3: https://osf.io/myxrz. This ad-hoc tool is composed of 12 items rated with a “meets the criteria” which will be assigned a positive sign (+), “does not meet the criteria” which will be assigned a negative sign (−) or, in case of doubt, a NS was assigned.

The risk of bias results was between 7 and 10 (lowest score: 1, and highest score: 12). No article was excluded based on the score obtained in this tool. The results of the bias analysis are available at Supplementary File 4: https://osf.io/596za. The inter-rater agreement obtainer in this analysis was 0.81.

### 2.5 Data extraction and management

The final studies included in the systematic review were coded in an Excel database by the first author. The coding was reviewed by the second and third authors, and any doubts were solved by discussion among all the authors. Information about study characteristics (authors, publication date, title, journal name, volume, issue and pages), method characteristics (study design, location, sampling, participants, number of participants, mean age and sex of participants), intervention details (intervention description, specific knowledge, attitudes or skills, comparisons, duration, intensity, number started, number completed, and conditions), outcome data (specific and collected, measurement instruments, validation status, duration of follow-up, and time of data collection), main conclusions, outcome-specific information (study limitations, possible publication bias, and imprecision of effect estimates), and finally, funding and possible conflict of interest. The codebook with the information extracted from the studies is available in Supplementary File 5: https://osf.io/s2xha.

## 3 Results

**Fig. 1:**
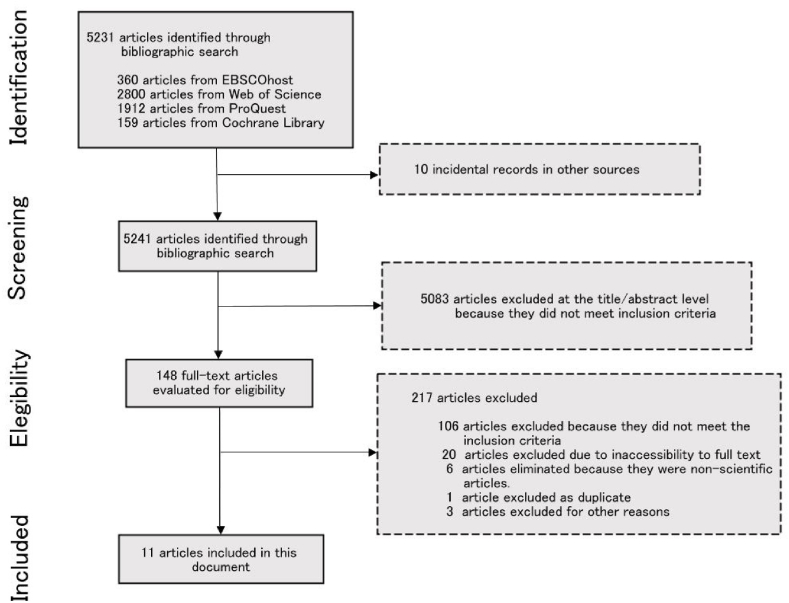
Flowchart of the study identiﬁcation process.

A PRISMA diagram detailing the study selection process is shown in Fig. 1. The electronic search yielded 5231 articles. 10 records were added for incidental bibliography, making a total of 5241 articles. After analyzing the title and abstract, 5083 publications were excluded because they did not meet the inclusion criteria. The full-text review of the remaining 148 articles resulted in the inclusion of 11 studies from which 38 strategies or proposals for user-directed interventions aimed at reducing workplace violence by users or their families toward professionals in the health sector were obtained.

### 3.1 General characteristics of the studies

The study characteristics are described in Table 1. Of the 11 studies located, the majority of the articles selected were qualitative studies [[24], [25], [26], [27], [28], [29], [30], [31], [32], [33], [34], [35], [36], [37], [38], [39]], 3 were randomized controlled trials [[40], [41], [42]], another study combined different methodologies, both qualitative and quantitative, throughout the implementation of the intervention [43], and one of them was a cross-sectional survey [44]. Most of the studies were conducted in the European continent (81.8%): Italy, United Kingdom, Sweden, Norway and Spain [[35], [36], [37], [38], [39], [40], [41], [42], [43], [44]]. The rest were carried out in Brazil and Australia [[34], [35], [36],^40^].

**Table 1:** Descriptive characteristics of the studies.

Study	Design	Country of study	n (number of participants)	Participants	Mean Age	Sex of the participants	STROBE
Henderson et al. [41]	Single-blind randomized controlled trial. Collects quantitative data.	London, United Kingdom.	80	Patients with Psychosis or bipolar disorder	Not specified	Not specified	9
Batista et al. [34]	Qualitative. Descriptive-exploratory. Collects qualitative data.	BELO HORIZONTE, MINAS GERAIS (Brazil)	Basic Health Units. Total of 12 groups: average of 7 people per group.	Nurses, doctors, managers, district directors, social workers, psychologists, psychiatrists, dentists, dental hygienists, assistants, nursing technicians, administrative staff, porters, general assistants, and users.	Not specified	Most of them are women	7
Ramacciati et al. [44]	Italian National Survey 2016. It collects quantitative and qualitative data.	Italy. 20 regions of Italy.	1100	Emergency Nurses.	Average age 42 ± 9 years	There were 144 women, 119 men, 2 undeclared	9
Fletcher et al. [40]	Cross-sectional survey study or RCT. Collects qualitative and quantitative data.	Victoria, Australia	72	Patients hospitalized in the psychiatric ward.	Public health service users.	52% [31] women 48% [29] men	10
O'Sullivan et al. [42]	RCT. Collects qualitative and quantitative data.	London	8 districts or wards.	Hospitalized forensic psychiatric service users and workers	Not specified	Not specified but men and women.	9
Pelto-Piri and Kjellin [35],	Qualitative	Sweden	33	Service users, staff members and room managers	Median 47 and mean 46.7 years	17 women and 16 men	8
Pina et al. [36]	Qualitative	Spain	80	Public health service users	Mean age 48.92 (SD = 14.95)	(63.7% female) (36.3% male)	9
Johnston et al. [37]	Qualitative	North England	30	Inpatients and forensic mental health staff members	Users: 18–43 years old. Health personnel:18–60 years old	18 women and 12 men	
O'Dowd et al. [38]	Qualitative	Scotland	7	Inpatients of a mental health hospital	Between 20 and 70 years old	100% men	
Faerden et al. [43]	Various phases: qualitative, collaborative, and quantitative work	Oslo, Norway	193	Users, family, professionals, and room designers (architects)	159 professionals: 32.7 years	Professionals: 50.75% Women 49.25% Men	
Pina et al. [39]	Qualitative	Spain	44	Primary Care Professionals.	Mean age: 50 years	68.14% women	

*Abbreviations*: RCT: Randomized Controlled Trial; SD: Standard Deviation.

Seven of the 11 studies included as participants users belonging to psychiatric units (63,6%) [[35], [36], [37], [38],[40], [41], [42], [43]]. Within that percentage, health care professionals were included [34,35,37,39,[42], [43], [44]]. Five of the studies included participants from both sectors, both users and workers [35,37,[42], [43], [44]]. However, in two other studies, the participants were all nursing staff [39,44] and, in four others, the participants were all users [36,38,40,41].

The mean age of the participants could not be collected in all the studies. Those in which it was collected (63.6%) ranged from 18 to 70 years of age. Almost all studies included both men and women, but female representation was slightly higher (63.6%). One of the studies did not specify the sex of the participants [39] and, in another study, the participation was exclusively male [38]. The total sample size was 1714 people. The number of participants was a minimum of 7 and a maximum of 1100.

#### 3.1.1 Duration of interventions and interviews

Of the results obtained, four of the studies were long-term interventions [[40], [41], [42], [43]]. Three of them ranged from 9 to 15 months [[38], [39], [40], [41], [42]] and the duration of the study of Faerden [43] was estimated at 5 years from the first phases of their qualitative study to the final evaluations, the evaluations were conducted during a period of 4 weeks before the implementation process and 3 months after the end of the implementation.

As for data collection in the qualitative studies, the duration of the interviews ranged from a minimum of 45 min to a maximum of 150 min [34,[36], [37], [38], [39]]. The online survey results of Ramacciati's work [44] were collected over a prolonged period of time (8 months). For the qualitative study by Pelto-Piri and Kjellin [35], it was not possible to determine how long the social insertion measures had been in place when the data collection took place.

#### 3.1.2 Classroom studies with long-term final evaluation

For the Henderson study [41], a treatment preference plan was drawn up jointly by users of the mental health service and professionals. With the permission of the users, after the review and approval of both parties, and an external staff as mediator, it was distributed among the emergency services to which the user could go and thus the user's preferences could be followed in situations in which they had difficulty expressing them. This shared decision-making was followed up immediately and again 15 months after its implementation by all those involved, both users and professionals.

The implementation of Safewards is both a model and a set of interventions designed to improve consumer and staff safety, and this method was followed in the study of Fletcher [40]. A post-intervention survey was administered to patients who had been hospitalized on the service for 1–4 weeks, although the Safewards measures had begun to be implemented 9–12 months earlier. Data were collected over 3 months. Some of the measures implemented within the Safewards package were collected for this review and aimed to strengthen a sense of shared community, a sense of common humanity, strengthen confidence and skills in the face of distress, and generate hope.

The main objective of Faerden's [43] work was to improve the dignity of patients, and thus the isolation and aggression, by transforming the environment and structure of psychiatric wards. First, a qualitative study was conducted with users and family members in order to gather proposals for improvement. In the next phase (design phase), collaborative work teams were formed between users, professionals and designers. This was followed by the remodeling phase of the rooms aimed at creating more welcoming environments with greater privacy and contact with nature. Evaluations of the results through a questionnaire were collected at two key points of the process, during 4 weeks before the implementation and 3 months after the remodeling.

#### 3.1.3 Long-term on-site studies with continuous assessment

One of the included articles had a long-term follow-up of the intervention on an ongoing basis. The intervention used by O'Sullivan [42] consisted of a package of improvements that included identifying the problem, analyzing its causes and creating a theory of change, Plan-Do-Study-Act, testing ideas and evaluating their impact on the system at regular intervals. Thus, if the measures applied were successful, they were incorporated into the program, and if not, they were eliminated from the program. These were collected through joint meetings by all involved and data collected over 15 months. It sought to foster a culture of openness within the organization around violence and to help service users and staff work together to understand and address it. This innovation sought to empower service users to take ownership and become more actively involved in reducing violence and aggression in the wards.

#### 3.1.4 Short-term qualitative studies

There were 6 studies that opted for a methodology using focus groups guided by experts. Batista et al. [34] sought to investigate and understand the violence experienced in Basic Health Units (UBS) from the perspective of work processes. The interviews with both professionals and users lasted between 90 and 150 min. Some intervention measures aimed at the user and links with the community were analyzed.

The participants, users, and staff, in Pelto-Piri and Kjellin's research [35], were also subjected to focus group analysis whose objective was to analyze violence around social inclusion through the analysis of three values: participation, reciprocity and social justice. The interviews with users lasted approximately 50 min. Interviews with staff and managers lasted between 60 and 90 min. The main question for users was about perceptions of feeling safe or unsafe in the ward and the main questions for staff and managers were about values at work in the interaction with the user, general safety questions and the handling of situations that were violent or presented a risk of violence.

The duration of the focus groups for the studies by Pina et al. [36] and Pina et al. [39] was between 60 and 70 min, aimed to analyze the existing sources of conflict in Primary Care centers and their possible solutions from the point of view of the user exclusively [36] and the professionals respectively [39]. All participants were asked questions related to violent situations in primary care centers. These questions were divided into three blocks: those related to the organization itself, those related to the professionals and those generated by the users themselves. They were then also asked about the solutions they provided to these conflicts and the users' perception of the changes following the emergence of COVID-19.

Five focus groups and one individual interview were conducted with an average duration of 67 min and 38 min for the interview in Johnston's study [37]. Both professionals and users participated in them with the aim of identifying and describing those barriers and facilitating elements for the improvement of de-escalation techniques.

Users in the O'Dowd study [38] participated in semi-structured interviews about the users' experience and knowledge of managing and assessing the risk of violence in a low-risk psychiatric ward. None of the interviews lasted more than 45 min.

#### 3.1.5 Online studies

Ramacciati et al. [44] distributed online a unique validated 39-item questionnaire on workplace violence among accident and emergency department workers. Within that survey, an open-ended qualitative survey was conducted on perceptions of verbal and physical violence in the workplace, where professionals proposed some measures taken into account in this research. The questionnaire was kept online for 8 months.

### 3.2 Outcome measures

The outcome measures for the different studies vary depending on whether they were quantitative or qualitative data, or even a combination of both.

In Henderson's study [41], an immediate follow-up was made and another one 15 months after the intervention plan was implemented. The same questions were asked to both users and workers in both cases. In the immediate follow-up, quantitative data were collected using a 5-point Likert scale and free text, while in the long-term follow-up, an 8-question Likert scale questionnaire was used.

Several studies analyzed both quantitative and qualitative data simultaneously [42,44] although they did not follow the same methods of analysis.

O'Sullivan et al. [42] analyze their quantitative data collected taking into account as an outcome measure the incidence rate of inpatient violence and aggression per 1000 occupied bed-days. The qualitative data were collected in routine meetings and an established “Improvement Model” methodology was used [45].

For three other studies, data were collected by quantitative analysis through a survey and qualitative analysis through open-ended questions [40,44]. For the work of Ramacciati [44], a 38-item online survey was conducted using a QuIN16VIPs questionnaire [46], the qualitative data were analyzed by inductive analysis [45], which have been of interest for this research. For the study by Faerden et al. [43] it was not possible to determine the method of qualitative analysis during the discovery phase with users and their families. However, for the evaluation phase, they asked 2 questions using a Likert scale at two key points in the process, before and after the remodeling of the room. A descriptive analysis was performed in Fletcher et al. [40] and the qualitative data were analyzed with an inductive and constructionist approach using six-step thematic analysis outlined by Braun and Clarke [47]. The latter data analysis was also shared by the work of Pina [36] and Pina [39] for their qualitative study. In the study by Peltro-Priri and Kjellin [35] and Johnston [37] the Framework method was used [48].

In the work of Batista et al. [34], results were collected after 12 months of implementation of some preventive measures. The results were also analyzed by qualitative analysis according to the method proposed by Bardin [49]. The answers to the open-ended question were analyzed using Van Kaam's method [50]. This in turn served to collect future proposals that have also been taken into account as results in this review.

Finally, for the semi-structured interviews in O'Dowd‵s [38] qualitative study, the steps suggested by Smith et al. [51] for IPA analysis were loosely followed [51].

### 3.3 Interventions or proposals collected

Given that the objectives of this review were to collect both interventions and proposals aimed at the user, the results show that, of the 11 articles included in this review, 8 analyzed measures or interventions aimed at reducing violence that were either newly implemented or intended to improve measures already in place [[34], [35], [36], [37], [38],[40], [41], [42], [43]] and 3 proposed future measures for the same purpose [38,39,44].

For the works of Henderson et al. [41], Johnston [37], Faerden [43] and O'Dowd [38], all the proposals put forward in the studies were collected. For the study by Batista et al. [34], a total of four categories were identified: individual strategies in the face of violence, institutional responses and support mechanisms, consequences and impacts of violence, and preventive actions. Of these, and consistent with the objectives of this review, four measures were recorded in our study. Ramaccití's research [44] suggested 27 topics that were grouped into four main blocks: perception of aggression, triggering factors, consequences, and solutions. From this last block, 2 measures of interest for the present review were selected.

The package of measures implemented in the Saferwards program consisted of 10 different interventions, 4 of which were identified as eligible [40]. The initial intervention by O'Sullivan et al. [42] consisted of a package of changes with three interventions: safety groups, weekly discussions, and safety crossovers. In the course of collaborative learning, user-led meetings were added. Only one was discarded because it was not user-driven, making a total of 3 measures coinciding with our objectives. Pelto-Piri and Kjellin [35] in their qualitative study compiled 5 main themes: patient participation in treatment and care, sense of community, us and them, access to good care, and quality of care. Six of the proposed measures were of interest. The qualitative user research by Pina et al. [36] managed to record a total of 4 thematic blocks for the identification of conflicts and 5 thematic blocks for the proposal of solutions to them. Of all the proposals, 10 were considered user directed. Likewise, in the qualitative study of professionals, five measures eligible for this study were extracted from the two thematic blocks of proposals for improvement of the identified sources of conflict [39].

All the studies included in this review adopted different approaches to combat workplace violence. However, the different approaches can be grouped into different categories according to the objectives pursued: improving communication and the creation of links between users and professionals, improving user information and training, involving the user in joint decisions with staff, support groups for users, and other types of individual objectives.

Therefore, a total of 38 interventions and/or proposals for user-directed interventions aimed at reducing violence and/or improving the relationship between the user and health professionals were collected. Table 2 and Table 3 show the list of more detailed results according to objectives. In general, 14 of the measures were aimed at improving communication and the creation of links between users and professionals [[34], [35], [36],^40^,^41^,^43^], thirteen of the proposals were aimed at improving information and training of users [34,35,39,46], on the other hand, six of the 38 proposals decided to involve the user in joint decisions with the staff [35,37,38,[40], [41], [42]]. Likewise, three proposals from two different studies advocated the creation of user support groups [36,39]. Finally, we found that one of the measures focused on strengthening communication among the users themselves [40] and another on making changes in the physical environment and environmental changes in order to improve the users' well-being [43].

**Table 2:** List of interventions/proposals according to objectives.

Intervention proposal	References
Improve communication and the creation of links between users and professionals.	
Ticket exchange between workers and users	Batista et al. [34]
Improve communication strategies	Batista et al. [34]
Community representatives at health centers	Batista et al. [34]
Create a greater sense of community	Pelto-Piri and Kjellin [35]
Patient participation in care and treatment	Pelto-Piri and Kjellin [35]
Improve the sense of community	Pelto-Piri and Kjellin [35]
Us and them	Pelto-Piri and Kjellin [35]
Improved quality of care	Pelto-Piri and Kjellin [35]
“Safe rooms” program: meetings and patient assistance.	Fletcher et al. [40]
Program: “Safe Rooms”: Get to Know Each Other	Fletcher et al. [40]
Safety crossings	O'Sullivan et al. [42]
Weekly community meetings	O'Sullivan et al. [42]
Humanization of Primary Care	Pina et al. [39]
Work teams	Pina et al. [39]
**Improved user information and training**	
Inform and educate the population	Batista et al. [34]
Information and educational campaigns for users	Ramacciati et al. [44]
Improving the image of nursing staff through the press	Ramacciati et al. [44]
Promoting health education.	Pina et al. [36]
Training in the use of new technologies	Pina et al. [36]
Medical care/information provided to the user	Pina et al. [36]
Information on waiting time/delay in your appointment schedule	Pina et al. [36]
User awareness campaigns	Pina et al. [36]
Mental health user campaigns	Pina et al. [36]
Preventive measures	Pina et al. [36]
Setting boundaries	Pina et al. [36]
User education	Pina et al. [39]
Welcome plan	Pina et al. [39]
**Involve the user in joint decisions with the staff.**	
Joint Crisis Plan	Henderson et al. [41]
“Safe rooms” program: Mutual clarification of expectations	Fletcher et al. [40]
Enhancing patient participation in care and treatment	Pelto-Piri and Kjellin [35]
Safety meetings led by service users	O'Sullivan et al. [42]
Improved de-escalation technique	Johnston et al. [37]
Violence risk assessment and management plan	O'Dowd et al. [38]
**User support groups**	
Health care by support groups	Pina et al. [36]
Psychosocial Support	Pina et al. [36]
Group therapy	Pina et al. [39]
**Isolated proposals**	
“Safe rooms” program: downloading of messages	Fletcher et al. [40]
Physical and environmental changes of the room	Faerden et al. [43]

**Table 3:** Characteristics of the intervention plans.

Intervention proposal	Study	What the intervention consists of	Effectiveness
Joint Crisis Plan	Henderson et al. [41]	Shared decision making, involving the patient in treatment decision making and in a crisis situation. Immediate and 15-month follow-up.	Quantitative data. Changes in the improvement of the relationship (46% in the immediate and 24% at 15 months), feeling of a better knowledge about his pathology (71% in the immediate and 56% at 15 months), improvement of the patient's feelings about himself (67% in the immediate and 48% at 15 months); He did not always resolve disagreements. Likelihood of continuing treatment was statistically significant from 36% to 3%. But users recommend it to other users (90%–82%).
Exchange of tickets between workers and users	Batista et al. [34]	Create a space for people to register their opinions, demands and complaints. Such an initiative is only effective if the criticisms are discussed as a team and, when necessary, with the community.	Proposed outcome of the focus group.
Improve communication strategies	Batista et al. [34]	Local meetings with the community. A qualitative study was conducted after 1 year.	A closer relationship with the community was achieved, strengthening the bond and the joint construction of alternatives to violence.
Community representatives at health centers	Batista et al. (343)	Community representatives at the health center, to enable the formation of links.	The qualitative study was done after 1 year with a representative: “when you listen to the community, things come to us that were not coming to us, in an objective way”.
Inform and educate the population	Batista et al. [34]	Informing the population about the operation of the Unified Health System and the Family Health Program Strategy.	Proposed outcome of the focus group.
Improving the image of nursing staff through the press	Ramacciati et al. [44]	Image of Accident and Emergency Nurses in the press to improve their image.	Suggestions from the qualitative question to nurses: “They never talk about our success stories, only medical malpractice and our mistakes”.
Information and educational campaigns for users	Ramacciati et al. [44]	We must educate citizens on how to properly use the Accident and Emergency Department.	Proposals for qualitative questioning of nurses.
“Safe rooms” program: meetings and patient assistance	Fletcher et al. [40]	In the daily meetings of professionals, shared daily meetings, the user attends and is given the option to request or demand help. Pick up between 1 week and 3 months in the ward.	It had a recall and acceptance of 81%. Acceptance of applicability 61%. 48 comments: 37 positive, 6 negative and 5 neutral. Reduction of almost 15% of conflictive events after the implementation of the “safe rooms” program. Greater recognition and respect. Greater sense of community.
Program: “Safe Rooms”: Get to Know Each Other	Fletcher et al. [40]	Builds rapport, connection, and sense of common humanity: Patients and staff share some personal interests and ideas with each other. Data were collected when patients had been on the ward for between 1 week and 3 months.	It had a recall and acceptability of 67%. Acceptability of applicability of 59%. 37 comments: 27 positive, 6 negative and 4 neutral. Reduction of almost 15% of conflictive events after the implementation of the “safe rooms” program.
“Safe rooms” program: Clear mutual expectations	Fletcher et al. [40]	Patients and staff work together to create mutually agreed-upon aspirations that apply to both groups equally. Data were collected when patients had been on the ward for between 1 week and 3 months.	It had a recall and acceptance rate of 33%. 36 comments: 24 positive, 6 negative and 8 neutral. Acceptance of applicability of 49%. Reduction of almost 15% of conflictive events after “safe rooms”. Variability among different staff members. Fairer expectations and greater respect.
“Safe rooms” program: downloading of messages.	Fletcher et al. [40]	Before discharge, patients leave messages of hope for other patients on a screen in the unit. Data were collected when patients had been on the ward for between 1 week and 3 months.	It had a recall and acceptability of 68%. Acceptability of applicability of 55%. 45 comments: 30 positive, 1 negative and 14 neutral. Reduction of almost 15% of conflictive events after the implementation of the “safe rooms” program. Feelings of hope. Difficulty in expressing themselves.
Safety crossings	O'Sullivan et al. [42]	Staff applied colored sticky dots (according to violence-free days or type of incident) on a kind of calendar visible to all (users and staff) to represent whether an hour or shift was incident-free (green). Duration 15 months.	Both quantitative and qualitative data. achieved and maintained an 8% reduction in incidents of physical violence and a 16.6% reduction in non-physical violence per 1000 occupied bed-days Helped to jointly celebrate achievements and maintain enthusiasm and momentum.
Weekly community meetings	O'Sullivan et al. [42]	Service users were encouraged to discuss with reference to cross security data. Staff ensured that discussions were non-judgmental. Service users were asked to reflect on the emotional impact of such events. Duration 15 months.	Both quantitative and qualitative data. an 8% reduction in incidents of physical violence and a 16.6% reduction in non-physical violence per 1000 occupied bed-days was achieved and maintained. They helped to identify frustrations and conflicts, analyze the causes and antecedents of violence, understand environmental factors and adapt them to the context.
Safety meetings led by service users	O'Sullivan et al. [42]	User-led meetings aimed at empowering service users to take ownership and become more actively involved in reducing violence and aggression in the wards. Duration 15 months.	Both quantitative and qualitative data. an 8% reduction in incidents of physical violence and a 16.6% reduction in non-physical violence per 1000 occupied bed-days was achieved and maintained.
Enhancing patient participation in care and treatment	Pelto-Piri and Kjellin [35]	Involve patients in care and treatment.	Results of the qualitative study express they had difficulties in involving patients, especially if they had been in the service for a long time. Establishing joint plans and listening to the user seems to be motivating.
Create a greater sense of community	Pelto-Piri and Kjellin [35]	Promote the room as a meeting place.	Results of the qualitative study state: forming relationships between staff and service user allows staff to recognize the relationships of service users better and the user protects staff if they see that they cannot control them. Other times service users did not perceive the approach by staff.
Patient participation in care and treatment	Pelto-Piri and Kjellin [35]	Encourage sensible communication between patients and staff.	Results of the qualitative study state: Both staff and users noticed improvement after trying to listen to the user and taking into account their preferences.
Improve the sense of community	Pelto-Piri and Kjellin [35]	The patient community as a resource: encouraging users to help each other.	Results of the qualitative study express: Encouraging users to help each other seems to be well received although they also comment that sometimes certain users can cause anxiety to others.
Us and them	Pelto-Piri and Kjellin [35]	The sanitary hierarchy, the hard jargon. Improve the way of relating, closer, improve the language.	Results of the qualitative study state: There was a perceived high tolerance for verbal threats, it was important to all that both staff and users did not use language that was not tolerated by society. All parties involved felt that psychiatry was evolving in the right direction: more towards humane attitudes and less towards hierarchization.
Improved quality of care	Pelto-Piri and Kjellin [35]	Minimize coercion, violence and injury and improve communication.	Results of the qualitative study express: They reported that as a result of communication they minimized coercive measures. They noted improvements in handling difficult incidents which reduced both staff and user injuries.
Promotion of health education	Pina et al. [36]	Promoting health education with the aim of reducing appointment seeking through self-care	There are no measures of effectiveness. This is a proposal for a qualitative user study.
Information on waiting time/delay in your appointment schedule	Pina et al. [36]	Inform the patient of the actual consultation time, either through an application, a message or in the waiting room.	There are no measures of effectiveness. This is a proposal for a qualitative user study.
Health care by support groups	Pina et al. [36]	Health care through support groups for users with the same pathology in order to improve care times.	There are no measures of effectiveness. This is a proposal for a qualitative user study.
Training in the use of new technologies	Pina et al. [36]	To train the user in the use of new technologies available in the service.	There are no measures of effectiveness. This is a proposal for a qualitative user study.
Medical care/information provided to the user	Pina et al. [36]	Improvement of the medical care/information provided to the user by the professionals.	There are no measures of effectiveness. This is a proposal for a qualitative user study.
Psychosocial support	Pina et al. [36]	Creation of support groups to solve psychosocial problems: relaxation and emotional management techniques.	There are no measures of effectiveness. This is a proposal for a qualitative user study.
Preventive measures	Pina et al. [36]	Increase preventive measures focused on more integrative medicine.	There are no measures of effectiveness. This is a proposal for a qualitative user study.
Setting boundaries	Pina et al. [36]	Set boundaries to the conflictive user without punishment, with consequences.	There are no measures of effectiveness. This is a proposal for a qualitative user study.
User awareness campaigns	Pina et al. [36]	Targeted user awareness campaigns on violence using social networks and others	There are no measures of effectiveness. This is a proposal for a qualitative user study.
Mental health user campaigns	Pina et al. [36]	Post-pandemic user-directed mental health campaigns.	There are no measures of effectiveness. This is a proposal for a qualitative user study
Improved de-escalation technique	Johnston et al. [37]	Set of actions to improve the de-escalation technique with greater user involvement: collaborative planning of de-escalation between professionals and patients, joint medication decisions and use of sensory rooms and voluntary seclusion.	There are no measures of effectiveness. It is a proposal for a qualitative study of users and professionals.
Management and evaluation plan	O'Dowd et al. [38]	Collaboration of users together with professionals in their management plan and assessment of the risk of violence.	Risk assessment was often perceived by the user as a tool at the service of professionals and not for the benefit of the user. Lack of perception of collaboration. Distant treatment. Users perceived a greater sense of responsibility on the part of the user and commitment on the part of the professional.
Physical and environmental changes in the room	Faerden et al. [43]	Changes in the physical environment: warm colors, decoration reminiscent of nature, single rooms with bathroom and closet, location promoting silence and smoking space.	Linear mixed model analysis showed a significant increase over the control group after changes in the physical environment for both professionals and users of 1.38 and 1.2 respectively.
User-directed education	Pina et al. [39]	Promote the correct communication of the user's rights and duties, public health information and the use of emergency services.	There are no measures of effectiveness. This is a proposal for a qualitative user study
Group therapies	Pina et al. [39]	Group therapies with users to promote emotional and psychosocial education in PC.	There are no measures of effectiveness. This is a proposal for a qualitative user study
Welcome plan	Pina et al. [39]	Elaborate a welcome plan for people who are unfamiliar with the functioning of PC aimed at users.	There are no measures of effectiveness. This is a proposal for a qualitative user study
Work teams	Pina et al. [39]	To create collaborative work teams with representatives from all groups to improve communication with the community and strengthen ties.	There are no measures of effectiveness. This is a proposal for a qualitative user study
Humanization of PC	Pina et al. [39]	Measures aimed at improving humanization with the user, such as elimination of physical barriers, treatment in waiting rooms and care.	There are no measures of effectiveness. This is a proposal for a qualitative user study

### 3.4 Effectiveness of the proposals or interventions

Regarding the measures of effectiveness, three of the studies show quantitative data on the effectiveness of the intervention plans [[40], [41], [42], [43]], three others show qualitative data after the implementation of some preventive measure [34,35,38] and the rest were studies proposing measures or improvements collected as a result of their qualitative study [36,37,39] or as a result of surveys [44], without measuring effectiveness.

Of the 30 intervention proposals or measures collected, 20 of them are proposals and do not present measures of efficacy [[34], [35], [36], [37], [38], [39], [40], [41], [42], [43], [44]]. For four of the remaining measures, efficacy was assessed when users had been in the program for a short time (between 1 and 4 weeks) [40]. Four other measures were analyzed in the long term, at the end of the intervention plan [34,41], and the effectiveness of the last three was analyzed in the long term on an ongoing basis, during the program intervention [42]. For seven of the interventions, it could not be determined how long they had been in place at the time of data collection, but they measured the effectiveness of an implemented study using qualitative data [35,38]. Specific form characteristics of the above measures are shown in Table 3.

### 3.5 Positive effects of interventions

Table 3 shows the positive effects of the different measures. Of all the data collected after the implementation of the intervention plan, those aimed at improving communication and the creation of bonds between users and professionals concluded that there was a notable improvement in the user-professional relationship and that better bonds were created between the two [34]. They also state that protective bonds were established on both sides, that professionals respect users more if they know their preferences, that violent acts decreased as communication between users was enhanced, that less coercive measures were used, and that there was a feeling that psychiatry was evolving towards more humane attitudes and less hierarchization [35].

The qualitative data show that collaborative learning through weekly meetings was a strength of the project, helping to identify frustrations and sources of conflict, identifying the causes and antecedents to violence and adapting them to the context [42]. This influenced the reduction of injuries to both staff and users, improving their ability to handle violent incidents. Likewise, the quantitative data collected from the different studies show that a reduction of almost 15% in violent acts was achieved after the implementation of the set of measures [40] and a reduction of 8% in incidents of physical violence and 16.6% in non-physical violence [42]. Individually, the data collected within this communication improvement block had 37 positive comments and an acceptance of applicability of 61%. In addition, a recall and acceptance of 81% was observed for the measure “meetings and patient assistance” and in the case of “get to know the other”, 27 positive comments were obtained, with an acceptability and recall of 67% and applicability of 59%.

Interventions aimed at involving the user in joint decisions with the staff were not always successful. The development of a “joint crisis plan” was better received by professionals than by users. The clinicians and group holders state that they were not able to resolve disagreements, but, on the other hand, they consider that it was well received. In other words, users were willing to recommend the use of the service to other service users; this opinion hardly changed from the immediate follow-up (90%) to the delayed follow-up at 15 months (82%) [41]. However, they did perform better on other measures. For example, the “Clear mutual expectations” measure belonging to Fletcher's “safe rooms” program [40] was very well received, with 24 positive comments. Users emphasized that the measures were based on fairer expectations and with a higher level of respect for the user, had an acceptance among users of applicability of 49% and was a measure remembered by 33%. The comments of the study by Pelto-Piri and Kjellin [35], whose results show that the measure of “enhancing patient participation in care and treatment” seems to be a motivating element for the user, just as listening to the user is for the staff, are also positive. Some of the users in the O'Dowd study considered the “Violence Risk Assessment and Management Plan” as a key point for their recovery and education. They positively remarked that the joint collaboration with staff helped them to increase their sense of responsibility and they perceived a greater commitment to their recovery and expectations for the future.

In general, the quantitative results of Fletcher et al. [40], on users' recall and perceived acceptability, show that interventions that directly involved the user were better remembered. After the implementation of “safe rooms”, quantitative data show that 95% reported feeling safer, 85% felt more connected with staff and 70% reported improved “balanced position” between staff and users. The qualitative data obtained show that most of the interventions achieved a positive experience, with changes in the user-professional relationship, among the users themselves, and improvements in the expectations about the reality of the facility, as well as in the quality of the services provided.

Positive ratings were also obtained for the group of isolated measures. After the “Physical and environmental changes in the room” in Faerden's work [43], the workers interviewed felt that the changes in the environment took care of the patient's needs and met the needs of the staff. Linear mixed model analyses showed a large and significant increase of 1.38 and 1.2 respectively. There being no significant change for the score in the control group. No user opinions were collected after the “Physical and environmental changes in the ward”, so it is not possible to know the degree of effectiveness of these changes after the remodeling [43].

It was not possible to determine the positive effects for two of the objectives, improvement of information and user training [34,36,39,44], and the creation of user support groups [36,39], since they do not mediate effectiveness and were only proposals.

### 3.6 Negative effects of interventions

Disagreements or difficulties in the implementation of the different measures or interventions were also reported. The measure “Enhance patient participation in care and treatment” [34] expressed certain difficulties in involving those patients who had been in the service for a long time. There was also disagreement with the implementation of “Creating a greater sense of community” [35], certain users stated that they did not perceive a change in the approach of the staff. Likewise, in “Improving the sense of community”, users' help to each other may cause some anxiety on certain occasions [35].

In the elaboration of a “Joint Crisis Plan”, many users did not have the opportunity to implement it for the duration of the intervention because they were not in a crisis. As an alternative, it is proposed to elaborate it for daily situations. Some of those who did have the opportunity to do so stated that disagreements were not always resolved and that on some occasions their preferences were not taken into account due to medical indications. Ratings were higher at immediate follow-up than at 15 months. The users' probability of continuing treatment was statistically significant, from 36% to 3% in 15 months [41].

Batista et al. [34] reported that it is sometimes difficult to resolve conflicts due to the high burden of care. For the work of Fletcher et al. [40], some users felt that not all interventions are appropriate and respectful of consumers. Along these lines, in 6 of the 37 comments received on “mutual aid meetings”, users expressed that they did not see results and found them childish, and therefore doubted their usefulness. Likewise, for the “Clear mutual expectations” measure, some of the users expressed variability according to staff members, with full-time professionals having greater success. Staffing was also influential for the “getting to know each other” measure, as not all were willing to participate, and some users expressed discomfort with losing their privacy.

In one of the centers, after the implementation of the measures proposed by O'Sullivan [42], an initial reduction in violence was observed, but followed by an increase. The authors state that several factors influenced this increase: the admission of particularly defiant and aggressive patients, and because of staff changes and their unfamiliarity with the change package, the latter questioned its effectiveness and could influence the efforts and commitment of the rest.

There was a general feeling among interviewees of little participation in the “Violence Risk Assessment and Management Plan”. They commented that it was a measure that was done “to them” but not “with them” and, therefore, reinforced feelings of being judged. They highlighted the lack of close language that implied that they did not feel “active” part of the process, but rather a “receiver”. They did not feel involved in decision making, leaving them out of the process and feeling insecure and distrustful. They felt that their voices were not important, and even some users were unaware of this measure, and that there was collaboration with professionals for their management plan and assessment of the risk of violence [38].

## 4 Discussion

The results of the systematic review of 11 studies provide 38 different measures aimed at reducing workplace violence by users or accompanying persons towards healthcare professionals. All these measures are based on direct or indirect work with the users themselves. Although these studies presented different approaches, our results revealed that, for the most part, the measures were aimed at improving communication and creating links between professionals and users, improving information and training for users, involving users in decisions on their treatment or treatment of their pathology as a preventive measure, and creating user support groups. Finally, several isolated measures are included, such as encouraging the creation of links between the users themselves and physical and structural changes in the rooms to respect the user's dignity and thus reduce health violence.

Reinforcement of communication between professionals and users, aimed at favoring greater links that benefit the relationship, is the main theme of the measures included. These range from creating spaces to favor communication between both, such as participatory meetings, bulletin boards, focused efforts to increase communication techniques in both treatment communication and personal treatment, and other measures aimed at creating a greater sense of community. In 11 of the 14 interventions described in the results with proposed improvements in communication and bonding, it was possible to collect both quantitative and qualitative data on effectiveness. These measures helped, on the one hand, to reduce the rates of both physical and verbal violence and managed to strengthen the links between professionals and users thanks to the closer relationship. On the other hand, they reinforce listening to the community, so that the user felt more respected. The factors causing the aggression were better understood and the user felt more listened. These results coincide with the findings of Gudde et al. [52], where they investigated through a systematic review the experience and views of users on aggressive situations in mental health care, expressing the importance of good communication and the existence of a direct relationship between aggressive situations and the way in which the staff addresses patients. The problem is not the rules themselves, but the way they are applied and communicated to patients [52], language being a reinforcement tool with powerful potential [53]. The communication deficit has been picked up and expressed by users in numerous previous studies. In this regard, the user manifests the inadequate attitude of some professionals as a focus of conflict, highlighting the lack of initial greeting, scarce eye contact, lack of collaboration to resolve conflicts, depersonalized treatment, use of excessively technical language, feeling ignored, absence of detailed information on the therapeutic process and the feeling of lack of listening. These aspects are highlighted in the literature and focusing preventive measures on improving empathy, friendly language, improving assertiveness and courtesy prevent and reduce conflicts [[54], [55], [56], [57]].

One of the strong points of the proposed measures focuses on enhancing the information and training received by the user. The different proposals included in this review are focused on the need to improve information and training for the population on how the system works on its correct use, on its pathological processes, awareness campaigns on violence and more training for personal growth. Along these lines, previous studies point to the usefulness of raising awareness of the roles of healthcare workers and the need to provide workers with training courses to promote public health, burnout, and emotional stress [2]. Working on the initial reactions provoked by the lack of information and training may have greater effects on the reduction of workplace violence [54,58]. Somani et al. [1] conclude in their systematic review that the situations and circumstances that provoke these initial reactions must be impacted, as violence prevention training interventions directed toward staff bring about positive changes by increasing confidence and communication skills, yet are ineffective in decreasing rates of workplace violence [22,28,[59], [60], [61]]. This may be because changing staff behaviors has no effect on the behaviour of patients and their families.

In contrast, none of the measures included in our study measured effectiveness with respect to these objectives. However, many of the proposals in Pina's study [36] are related to information and training, a qualitative study whose opinions were exclusively those of the user, who also demand this need. Training of all parties involved seems to be the right way to prevent workplace violence in health care [56]. The lack of information is associated with the users' perception of a paternalistic attitude of certain professionals. Allowing the user to be an active manager of their own recovery and allowing them to take an active part in their health is well received by users [29,62,63]. This review includes several measures that share this objective. The participants remembered better those interventions in which they felt involved than those in which they did not, they made it possible to establish measures to deal with violence in a shared and joint manner between professionals and users, respecting and taking into account both parties, and improving knowledge of their pathology. Some previous studies highlight as a right the access to participate in decisions related to their health and the right to clear information [54]. The systematic review by Raveel and Schoenmakers [64] identifies as a risk factor for aggression the discrepancy between the user's expectations and their treatments. Involving the user could improve these discrepancies. Another factor associated with the good perception of patients regarding the health care received is waiting times and/or system saturation [36]. Although not included in our results, it would be interesting to explore the capacity of both professionals and the system itself to avoid overcrowding and/or possible conflicts, as is done in other areas of research [65].

The evidence shows that some of the measures proposed by the different qualitative studies with the aim of prevention, health promotion and psychosocial support, could have equally good results in their implementation, reducing the number of hospital admissions and improving the quality of care [[66], [67], [68]]. Another proposal focused on increasing the limits for the user; the bibliography proposes a culture of “zero tolerance”, creating rules and protocols of strict compliance by both professionals and users [[69], [70], [71]]. Likewise, the importance of respecting patients' rights and the relevance of including this in specific actions in prevention programs has also been pointed out [2].

Although the overall results of those interventions in which effectiveness could be measured are positive, to a lesser extent, the implementation of the measures also has drawbacks. Such as certain difficulties in following the measures implemented, either due to lack of participation of both the user and the professional, lack of motivation or discrepancy with the established norms.

### 4.1 Limitations

This study has limitations. Despite the effort by the research team to identify all potentially eligible studies through an extensive search of multiple databases and a variety of similar and related terms, it is possible that, especially in intervention programs (or proposals), there are studies that have not been published or that this research team has not been able to access. In addition, some of the studies collected did not report the effectiveness of their interventions, so we cannot determine their effect in reducing user violence in the health care setting. Among those that did measure effectiveness, we found that not all of them offered a long-term follow-up period, which hinders the effect of the proposals. Another limitation is the lack of clarity in the results of the studies, which do not specify in detail the length of stay of the patients.

### 4.2 Research and clinical implications

The following factors should be taken into account in planning future preventive measures: Following the recommendations of the systematic review conducted by Somani [1], which conclusively states that studies involving multicomponent interventions, policy changes, environmental changes and training were shown to have higher success rates in reducing violence rates than those that addressed only stand-alone training. However, they are the least well conducted, with only 5 of 26 studies including this package of changes in that review. Assuming this idea, it seems inevitable to think of new multi-action prevention models that include: staff training, user training, safety measures, structured prevention policies, workplace violence management and a package of user-directed measures [1,16,37].

Most of the systematic reviews conducted to date focus primarily on healthcare personnel, often neglecting direct work with users. These reviews usually deal descriptively with the consequences of aggression in personnel, or present intervention and training measures to prevent and minimize aggression in the workplace, but they are directed exclusively at health care workers [1,23,[72], [73], [74], [75], [76]].

We consider that what is proposed here serves as a basis for future studies. In the first place, we point out the need to carry out studies with adequate designs to evaluate the effectiveness of the programs, whether those described here, or others derived from them. In this line, our results allow the creation of programs with those aspects that are best suited to the context of each country, health system, unit (emergency, psychiatry, etc.) or professional group. Secondly, the aspects indicated show a wide range of variables related to violence in the work environment. Along these lines and following the proposal of the authors [77], the factors identified in the present study could serve as a basis for exploring explanatory models of workplace violence in the health care setting.

## 5 Conclusion

After reviewing the literature on intervention plans or strategies and proposals involving the user to minimize or prevent workplace violence in health care by users or their relatives against staff, we found that most of the measures are aimed at improving communication and creating links between users and professionals, followed by a package of measures aimed at improving information and training for the user. To a lesser extent, there are also strategies that involve the user in joint decisions with the staff on their treatments, pathological processes or treatment received, measures with the creation of support groups with users, and lastly there is a group of measures with characteristics that cannot be grouped together, such as greater communication between users themselves and a change in the structure and aesthetics of the rooms.

New prevention plans must go beyond the individual level and include a package of successful interventions that involve all stakeholders, users, professionals, and management. The implementation of measures at the user level is often neglected, and one of the purposes of this review is to raise awareness and work to reduce violence with actions that also involve the user. We believe it is necessary to introduce changes that promote communication, safety, trust, training, information, and user involvement if we want healthcare in safer environments. This set of measures provides researchers with a basis to consider for the implementation of future prevention plans. Further work is needed to improve them and to learn more about their effectiveness, and more studies are needed to measure the effectiveness of the joint programs.

## Declaration

### Ethics statement

This study was approved by the Research Ethics Committee of the University of the authors, University Miguel Hernandez (UMH) assigned the Code of the Office of Responsible Research (COIR) with ref. 220,426,115,743.

## Author contribution statement

1 - Conceived and designed the experiments;

2 - Performed the experiments;

3 - Analyzed and interpreted the data;

4 - Contributed reagents, materials, analysis tools or data;

5 - Wrote the paper.

## Declaration of competing interest

The authors declare that they have no known competing financial interests or personal relationships that could have appeared to influence the work reported in this paper.
