# Supplementary material for: User violence prevention and intervention measures to minimize and prevent aggression towards health care workers: A systematic review
Source: Heliyon. 2023 Sep 1;9(9):e19495. doi: 10.1016/j.heliyon.2023.e19495 (PMC10558594; doi:10.1016/j.heliyon.2023.e19495)
Supplement: Multimedia component 2 [file mmc2.docx]

**ANNEX I: Descriptors used for the different databases.**

( Health occupations or health profession* or health personnel or health person* or health care worker or health care workers or health care worker* or health care personnel or health care person* or health providers or health provider* or health care providers or health care provider* or health staff or healthcare staff or health care staff or health professionals or health professional* or healthcare professional* or health care professional* or health workers or health worker* or medical staff or medical personnel or medical person* or medical professionals or medical professional* or medical workers or medical worker* or medical providers or medical provider* or  clinician or nurs* or nursing staff or nursing assistant or patients or user or general practitioner or physician or primary health care professional* OR Profesiones sanitarias or personal sanitario or trabajador sanitario or trabajadores sanitarios or profesionales sanitarios or trabajadores de la salud or personal médico or profesionales médicos or médico or enfermer* or personal de enfermería or auxiliar de enfermería or pacientes or usuarios and Primary care or Primary health care or Primary healthcare or Health system or Emergency servic* or Mental Health or Intensive Care or ward clerk* or ward receptionist* or clinical assistant* or patient service assistant* or porter* or volunteer* or security ward* or security person* or security officer* or safety staff or health care security officer* or Admin* staff or admin* personnel or infraestructur* or Facilit* or Manag* or hospital staff OR Atención Primaria or Sistema sanitario or Servicios de Emergencia or Urgencias or Salud Mental or Cuidados Intensivos or Administración or Infraestructuras or Gestión or personal del hospital or seguridad or celador* or personal de limpieza or voluntario* ) AND AB ( user violence or user aggression or violencia de usuario or agresión del usuario ) AND AB ( Workplac* violence or violen* or aggression or aggression* or agress* or Hostility or hostil or inappropriate behaviour or harass* or disrupt* or incivility or abus* or assault* or emotional abuse or emotional violence or verbal abuse or verbal violence or sexual abuse or sexual violence or  physical agress* or  physical abuse or stress disorder or gender violence or disrupt* behaviour or patient agress* or workplac* aggress* or work-related violen* or injury or Violen* en el trabajo or violen* agresión o agresion* or agres* or hostilidad or hostilidad*  hostilidad or conducta inapropiada or acoso or incivilidad or abus* or asalto or abuso emocional-verbal or abuso emocional or violencia emocional or abuso verbal or violencia verbal or abuso sexual or violencia sexual or agresión física or abuso físico or estrés or violencia de género or conducta disruptiva or agresión del paciente or agresión en el trabajo or violencia relacionada con el trabajo ) NOT TI ( “Intimate partner violence” NOT “dating violence” NOT batter* NOT “violencia de género” NOT “violencia de pareja” NOT “violencia en el noviazgo” ) AND AB ( Organizational policy or policy or interven* policies or management or prevention or control or intervention plan* or interven* program* or prevent* plan or prevent* program or prevent* program* or prevent*strateg* or system chang* or plan or protocol or train* or safety measur* or risk assesment or risk* management or agress* management or course or formation or education program* or bio-psycho-social program* or biopsychosocial program* or bio-psycho-social model or biopsychosocial model or bio-psycho-social stand point perspective or biopsychosocial perspective or biopsychosocial approach or bio-psycho-social approach or biopsychosocial intervention* or bio-psycho-social intervention* or Política organizacional or política or política de interven*or manejo or administración or prevención or control or plan* de interven*or program* de interven* or plan* de preven*or program* de preven* or estrategia* de prevención or cambio* del sistema or plan or protocol*or formación or medid*de seguridad or curso or entrenamiento or formación or educación or educación biopsicosocial )
