# Supplementary material for: User violence prevention and intervention measures to minimize and prevent aggression towards health care workers: A systematic review
Source: Heliyon. 2023 Sep 1;9(9):e19495. doi: 10.1016/j.heliyon.2023.e19495 (PMC10558594; doi:10.1016/j.heliyon.2023.e19495)
Supplement: Multimedia component 3 [file mmc3.docx]

**ANNEX II. AD-DOC TOOL**

Scoring:

When the criterion is met 🡪 + (2)

When not met 🡪 - (1)

When it is not clear 🡪 NS (3)

**Assessment of the Method section:**

1. Participants:
   1. Cohort studies: provide eligibility criteria, as well as sources and method of participant selection. Specify follow-up methods.
   2. Case-control studies: provide eligibility criteria as well as sources and diagnostic process for cases and selection of controls. Provide the rationale for the choice of cases and controls.
   3. Cross-sectional studies: provide eligibility criteria and sources and methods of participant selection.
   4. Cohort studies: in paired studies, provide the criteria for pair formation and the number of participants with and without exposure.
   5. Case-control studies: in matched studies, provide the criteria for pair formation and the number of controls per case.
2. The variables of interest are clearly defined. The response format, predictor, confounder and effect modifier variables are also explained.
3. Data sources and details of the valuation (measurement) methods are provided. If there is more than one group, it is specified for each of them.
4. Specify whether measures against bias have been taken.
5. Specifies how the sample size was obtained.
6. Explain how quantitative variables were treated in the analysis. If applicable, explain which groups were defined and why.
7. Statistical methods:
   1. Specifies all statistical methods, including those used to control for confounding factors.
   2. Specifies all the methods used to analyze subgroups and interactions.
   3. Explain the treatment of missing data (missing data):
      1. Cohort study: if applicable, explain how losses to follow up are dealt with.
      2. Case-control studies: if applicable, explain how cases and controls were matched.
      3. Cross-sectional studies: if applicable, please specify how the sampling strategy is taken into account in the analysis.
   4. Describe the sensitivity analysis.

**Assessment of the Results section:**

1. Participants:
   - 1. Describes the number of participants in each phase of the study; for example: numbers of potentially eligible participants, those screened for inclusion, those confirmed eligible, those included in the study, those who had full follow-up, and those screened out.
     2. Describe the reasons for the loss of participants in each phase.
   1. Descriptive data:
      1. Describes the characteristics of study participants (e.g., demographic, clinical, social) and information about exposures and potential confounding factors.
      2. Indicates the number of participants with missing data for each variable of interest.
   2. Cohort studies: summarize the follow-up period (e.g., average and total).
2. Results variables:
   1. Cohort studies: describe the number of outcome events, or provide summary measures over time.
   2. Case-control studies: describe the number of participants in each category of exposure, or provide summary measures of exposure.
   3. Cross-sectional studies: describe the number of outcome events, or provide summary measures.
3. Main results:
   1. Provide unadjusted and, if appropriate, confounder-adjusted estimates and their precision (e.g., 95% confidence intervals). Specify the confounding factors for which adjustment is made and the reasons for including them.
   2. If you categorize continuous variables, describe the limits of the intervals.
   3. If relevant, consider accompanying estimates of relative risk with estimates of absolute risk for a relevant time period.

**Discussion Assessment**

1. The limitations of the study are discussed, taking into account possible sources of bias or imprecision. Both the direction and magnitude of any possible biases are reasoned.
2. Specified and non-conflicting sources of financing.
